# Vapor–Liquid Equilibrium Study of the Monochlorobenzene–4,6-Dichloropyrimidine Binary System

**DOI:** 10.1021/acsomega.2c00525

**Published:** 2022-05-19

**Authors:** Eniko Haaz, Daniel Fozer, Ravikumar Thangaraj, Milán Szőri, Peter Mizsey, Andras Jozsef Toth

**Affiliations:** † Environmental and Process Engineering Research Group, Department of Chemical and Environmental Process Engineering, Budapest University of Technology and Economics, Műegyetem rkp. 3, Budapest H-1111, Hungary; ‡ Division for Sustainability, Department of Environmental and Resource Engineering, Technical University of Denmark, Produktionstorvet, Building, 424, DK-2800 Kgs. Lyngby, Denmark; § Institute of Chemistry, Faculty of Material Science and Engineering, University of Miskolc, Egyetemváros A/2, Miskolc H-3515, Hungary; ∥ Higher Education and Industry Cooperation Center of Advanced Materials and Intelligent Technologies, University of Miskolc, Egyetemváros A/2, Miskolc H-3515, Hungary

## Abstract

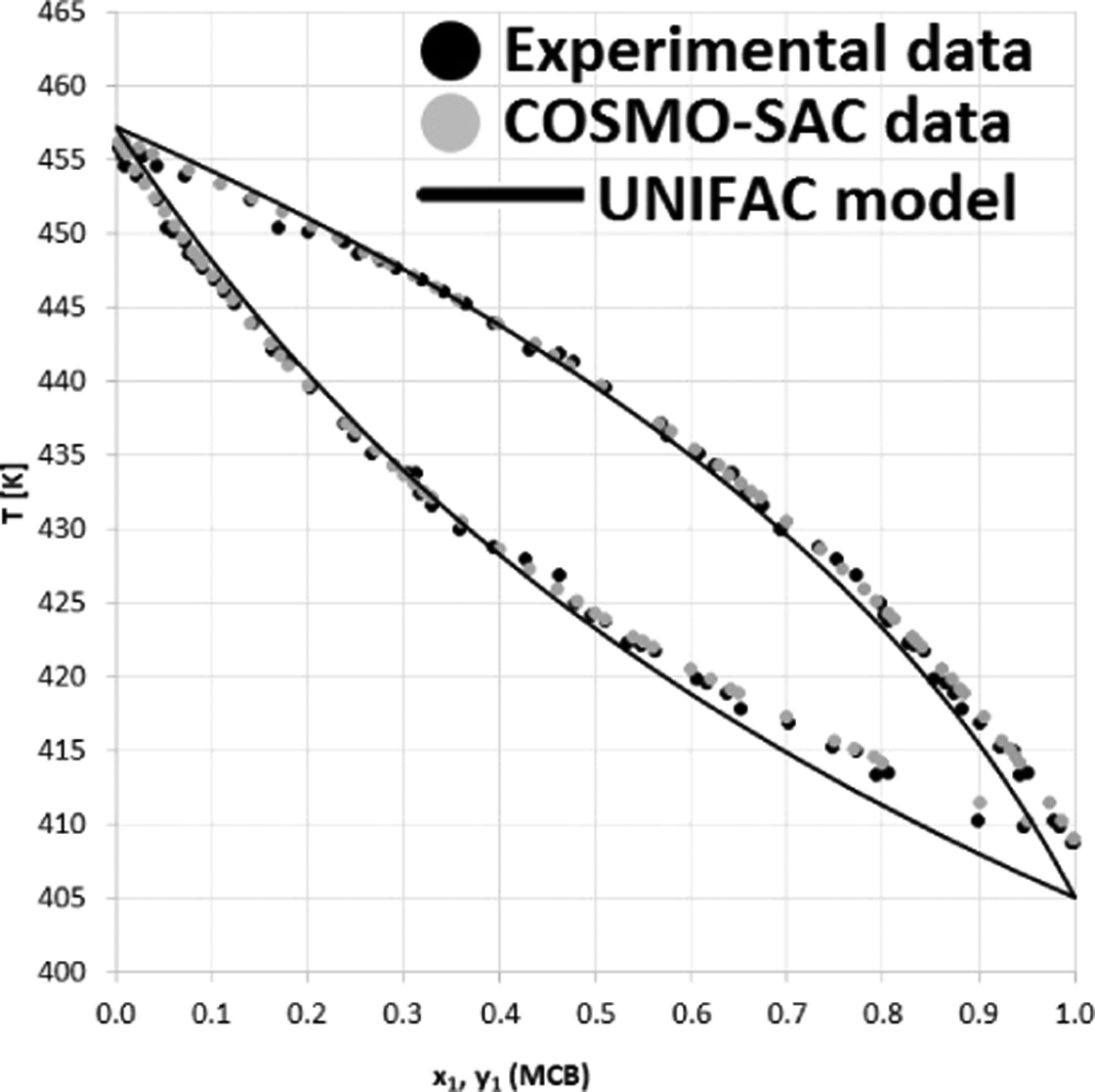

The number of newly synthesized and produced organic chemicals has increased extremely quickly. However, the measurements of their physical properties, including their vapor–liquid equilibrium (VLE) data, are time-consuming. It so happens that there is no physical property data about a brand-new chemical. Therefore, the importance of calculating their physicochemical properties has been playing a more and more important role. 4,6-Dichloropyrimidine (DCP) is also a relatively new molecule of high industrial importance with little existing data. Therefore, their measurements and the comparison with the calculated data are of paramount concern. DCP is a widespread heterocyclic moiety that is present in synthetic pharmacophores with biological activities as well as in numerous natural products. Isobaric VLE for the binary system of 4,6-dichloropyrimidine and its main solvent monochlorobenzene (MCB) was measured using a vapor condensate and liquid circulation VLE apparatus for the first time in the literature. Density functional-based VLE was calculated using the COSMO-SAC protocol to verify the laboratory results. The COSMO-SAC calculation was found to be capable of representing the VLE data with high accuracy. Adequate agreement between the experimental and calculated VLE data was acquired with a minimal deviation of 3.0 × 10^–3^, which allows for broader use of the results.

## 1 Introduction

4,6-Dichloropyrimidine (DCP) is an important compound as a starting material for medicines and pesticides. Cyclic voltammograms of 4,6-dichloropyrimidine show three cathodic waves arising from sequential cleavage of carbon–chlorine bonds as well as the reduction of pyrimidine ring^1^ pointing toward its chemically active sites. 4,6-Dichloropyrimidine was used in the synthesis of macrocyclic host molecules such as N-substituted azacalix[4]pyrimidines.^2,3^ Furthermore, DCP is also a starting reagent for the synthesis of disubstituted pyrimidines by tandem amination and the Suzuki–Miyaura cross-coupling, and it is used in a biarylpyrimidine synthesis involving biaryl cross-coupling as well.^4−6^

DCP is also used as a raw material for pesticides, for which high-purity DCP production is mandatory. However, the use of pesticides has its drawbacks. There is a risk of accumulating harmful chemical residues in plants, developing resistance to active substances, and destruction of useful pollinating insects in large numbers.^7^ To avoid these problems, the production of plant protection substances needs to be continuously improved, and one solution may be provided by sulphonylureas prepared from DCP and other aminopyrimidines.^8−10^

**Figure 1:**
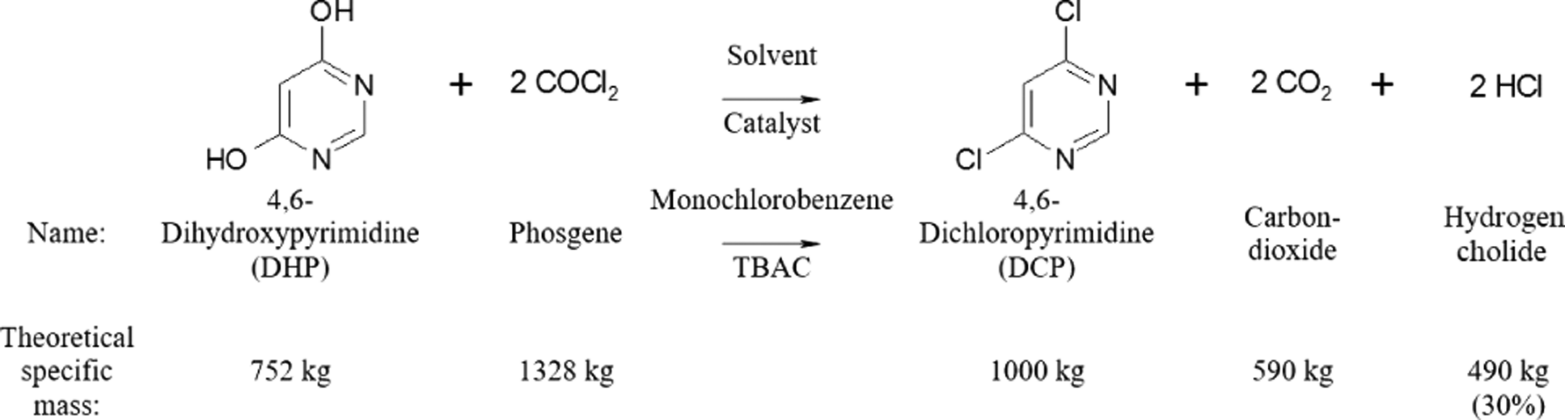
Phosgenation of 4,6-dihydroxypyrimidine to produce 4,6-dichloropyrimidine (DCP).

4,6-Dihydroxypyrimidine (DHP) is the starting material to produce DCP. The phosgenation of DHP proceeds in the presence of a monochlorobenzene (MCB) solvent and a tetrabutylammonium chloride (TBAC) catalyst. Carbon dioxide and hydrogen chloride are formed as byproducts in twice the stoichiometric amount^11,12^ as can be seen in Figure 1.

Technological steps include phosgenation, phosgene removal by nitrogen introduction, multiple distillation steps, and refining of the generated product.

For the preparation of DCP, monochlorobenzene solvent is used, which needs then to be recovered as efficiently as possible in the purification phase.^13,14^ To improve the distillation procedure, precise phase equilibrium data is required. Therefore, this research aims to determine the vapor–liquid equilibrium (VLE) of monochlorobenzene–4,6-dichloropyrimidine binary system, which has not yet been published in the literature.

UNIFAC and COSMO-SAC models were used for describing the equilibrium. UNIFAC thermodynamic model was first published by Fredenslund et al.^15^ The UNIFAC thermodynamic model for predicting liquid-phase activity coefficients provides the chemical engineer with a useful tool for calculating VLE compositions in the frequently encountered situation where no experimental information is available. The UNIFAC method is applicable to a wide range of systems exhibiting either negative or positive deviations from Raoult’s law. The method integrates the solution-of-functional groups concept with a model for activity coefficients based on an extension of the quasichemical theory of liquid mixtures (UNIQUAC). The UNIFAC thermodynamic model provides a simple procedure for calculating activity coefficients in terms of constant reflecting the surface areas and sizes of individual functional groups and parameters representing energetic interactions between groups. Size and area parameters for groups are evaluated from pure-component, molecular structure data.

Gmehling et al.^16^ modified the UNIFAC method. The main advantages of the modified thermodynamic model were the real behavior in the dilute region a better description of the temperature dependence. It has also become applicable for mixtures involving molecules very different in size.

## 2 Materials and Methods

The accuracy is quite a critical issue in the vapor–liquid equilibrium measurements to obtain high-quality data. Accurate measuring devices such as thermometers, analytics, and pressure meter should be applied to exclude systematic errors. Since the pressure influences the vapor–liquid equilibrium dramatically, special attention needs to be paid to provide the ambient pressure when measurements are taken. To do so, we applied high-quality and tested measuring devices like a thermometer, pressure meter, and Shimadzu GC/FID for analytics.

The properties of the chemicals used in the present work are introduced in Table 1. Monochlorobenzene and 4,6-dichloropyrimidine were purified by vacuum distillation at *P* = 13 kPa. The organic content was measured with a Shimadzu GC2010Plus+AOC-20 autosampler gas chromatograph with an HP-5 (30 m × 0.32 mm, 0.25 μm) column connected to a flame ionization detector using hydrogen as carrier gas. One hundred microliters of the samples were taken separately from the steam and liquid samples and homogenized with 1 mL of dichloromethane and injected into the apparatus. From the obtained chromatograms, concentrations were obtained based on the area ratio of MCB to DCP. The conditions of GC analytics and a sample chromatogram can be found in the Supporting Information.

**Table 1:** Description of Chemicals Applied in This Work (MW: Molecular Weight)

	CAS Reg.	formula	suppliers	initial mole	purification	final mole	analysis
component	No.			fraction purity	method	fraction purity	method
monochlorobenzene (1)	108-90-7	C_6_H_5_Cl	Sigma-Aldrich	0.9980	distillation	0.9995	GC
4,6-dichloropyrimidine (2)	1193-21-1	C_4_H_2_Cl_2_N_2_	Sigma-Aldrich	0.9700	distillation	0.9990	GC
acetonitrile (1)	75-05-8	C_2_H_3_N	Sigma-Aldrich	0.9980	distillation	0.9995	GC-MS
water (2)	7732-18-5	H_2_O	Sigma-Aldrich	0.9999	none		

**Figure 2:**
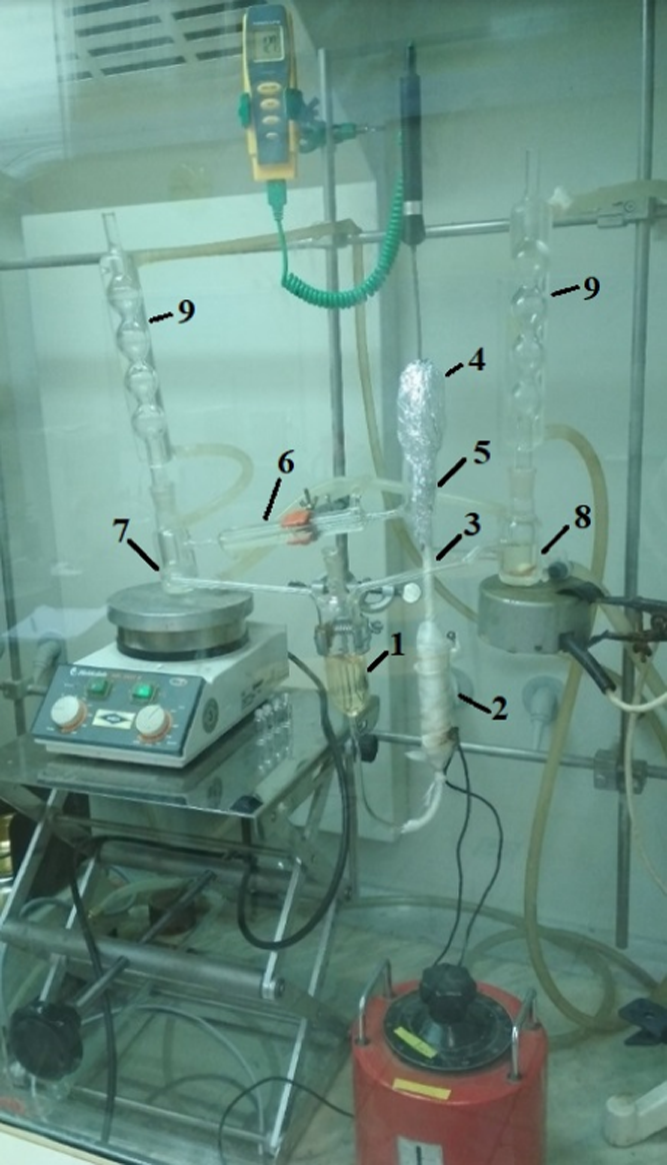
VLE experimental apparatus (1, liquid container; 2, boiler tube; 3, Cotrell pump; 4, thermometer well; 5, equilibrium chamber; 6, vapor condenser; 7, vapor sampler; 8, liquid sampler; 9, condensers with vacuum connections). The photograph was taken by Andras Jozsef Toth. Copyright 2022.

The vapor–liquid equilibrium experiments were achieved with a modified Gillespie apparatus.^17^ Duplicated sampler walls on the unit were applied to establish external cooling to decrease casual evaporation of the components from the liquid and vapor samples. Powerful mixing of the samples was achieved by magnetic stirrers in both sampler parts. The temperature was examined with a digital thermometer (223–573 K) with an accuracy of 0.1 K. The atmospheric condition was measured with an uncertainty of 2 kPa.^18^ The VLE experimental apparatus can be seen in Figure 2.

To circulate the mixture in the device continuously, 80–100 mL of liquid is required, which can be filled via the liquid sampler (8) after the condensers is removed. The sample to be measured drips from here into the liquid container (1). At least enough samples are needed for the liquid in the boiling tube to reach the Cotrell pump. The inner wall of the boiling tube was coated with glass powder to promote nucleation and prevent better heating and overheating. The heating was controlled with a toroidal transformer. Stirrers were placed in samplers (7 and 8) and the equilibrium phases were homogenized using a magnetic stirrer, since a uniform concentration in the sampling units is vital for obtaining reliable vapor–liquid equilibrium data.

Water cooling was used to cool the equipment, which provided cooling of the mixture in the condensers and double-walled samplers. Upon heating, the liquid begins to boil and the resulting vapor–liquid mixture reaches the Cotrell pump and then enters the thermometer housing where the equilibrium temperature can be measured. The liquid then enters the equilibrium chamber (5), where the two phases are separated. The liquid phase drains into the liquid sampler (8) and the steam generated condenses on the outer wall of the equilibrium chamber and then flows into the vapor sampler (7). When there is already a larger amount of liquid in the tanks, they flow back into the liquid tank and thus circulate in the device. Due to the cylindrical design of the liquid tank, no liquid flowed back into the sampling space during the level fluctuation.

Equilibrium can be determined by continuous monitoring of the temperature. In the stationary state, the temperature does not change, in which case, sampling takes place. After reading the temperature, first the liquid and then the steam condenser is removed and a sample is taken from that phase using an automatic pipette. Between two samples, it usually takes 20–25 minutes for the mixtures to reach a permanent stationary state. Sampling takes place when the temperature does not change for a minimum of 5 minutes.

After sampling, the composition of the mixture can be easily modified by adding one of our pure substances to the liquid in the liquid-side container, depending on the direction in which we want to shift the equilibrium. The collection of the vapor–liquid equilibrium data pairs should always be started by circulating the pure (usually more volatile) compound so that the purity of the device can be checked as mentioned earlier, and the continuous addition of the less volatile compound can accurately measure the equilibrium data over the entire concentration range.

For analyzing the equilibrium sample of acetonitrile–water, the refractive indexes were determined. As a validation for the refractometric method, GC analysis was performed for calibration samples and certain equilibrium.^18^ A Carl Zeiss Abbe Refractometer (Type G) was applied for the analysis of refractive indexes. The accuracy of the refractometer is 0.0001 according to the manufacturer at 293.2 K. Experimental and literature refractive indexes (*n*_D_) and Antoine constants (*A*, *B*, and *C*) of the applied chemicals are introduced in Table 2. It can be stated that the experimental refractive indexes show good agreement with the literature data.

**Table 2:** Experimental and Literature Refractive Indexes (*n*_D_) at 293.2 K of Pure Compounds Used and Their Antoine Constants (*A*, *B*, and *C*) and Validity Temperature Range

property	acetonitrile	water
*n*_D_ present work	1.3435	1.3320
*n*_D_ literature	1.3421	1.3329
*n*_D_ reference^a^	(19)^a^	(20)^a^
Antoine constants^b^		
*A*	4.27873	5.08354
*B*	1355.374	1663.125
*C*	–37.853	–45.622
*T*-min/K	288.3	344
*T*-max/K	362.3	373
reference	(21)	(22)

a Standard uncertainty: *u* is *u*(*n*_D_) = 2% approximately (acetonitrile) and *u* is *u*(*n*_D_) = 0.0003 (water).

b Antoine constants (bar, K) of acetonitrile and water were calculated by NIST from literature data.

The composition of monochlorobenzene–4,6-dichloropyrimidine binary system was measured with the above-mentioned Shimadzu GC2010Plus+AOC-20 autosampler gas chromatograph.

At a given temperature (*T*) and pressure (*P*), the equilibrium composition of the vapor (*y_i_*) and liquid (*x_i_*) can also be estimated accurately from the first principles calculation according to Klamt’s conductor-like screening model (COSMO^23−25^). This COSMO procedure links the microscopic surface-interaction energies and the macroscopic thermodynamic properties of a liquid via statistical thermodynamics. In COSMO calculations, a molecule separates into several parts called segments and charge distributions over entire segments are calculated to neutralize the whole molecule. Location of segments, segment areas, and charge densities are the computed properties. To perform COSMO-SAC calculations, surface area (*A*) and cavity volume (*V*) of the molecule, location of segment (a vector with *x*, *y*, and *z* coordination), and its charge density and area (*An*(σ)) are generated. In this approach, all molecular interactions consist of local pairwise interactions of surface segments. The statistical averaging can be done in the ensemble of interacting surface pieces. To describe the composition of the surface-segment ensemble with respect to the interactions, only the probability distribution of polarization charges (σ) must be known for all compounds (σ-profiles). The σ-profile of the whole system/mixture is just a sum of the σ-profiles of the components weighed with their mole fraction. The chemical potential of a surface segment with screening charge density σ, which is called σ-potential, is an implicit function of the polarity σ and therefore must be solved iteratively. The partial Gibbs free energy of a compound in a system of interest is readily available from the integration of the σ-potential over the surface of the compound. This is temperature-dependent, which can then allow us to predict almost all thermodynamic properties of compounds or mixtures. In our case, the *T*–*y*–*x* diagram had been calculated at the total pressure of 101 kPa.

In this work, compounds of interest have only one conformer in the absence of flexible groups; therefore, only one initial structure for each molecule was generated and used for BP/def-TZVPD(Fine) gas-phase geometry optimizations and energy calculations of the condensed-phase geometries conducted by the ADF program package.^26^ Then, the results from BP/def2-TZVPD-FINE calculation were used in COSMO-SAC (segment activity coefficient) calculations as implemented in the ADF program.^27−29^ Furthermore, for benchmarking purposes, the conventional UNIFAC model^16^ was also applied for the monochlorobenzene–4,6-dichloropyrimidine binary system.

## 3 Results and Discussion

**Figure 3:**
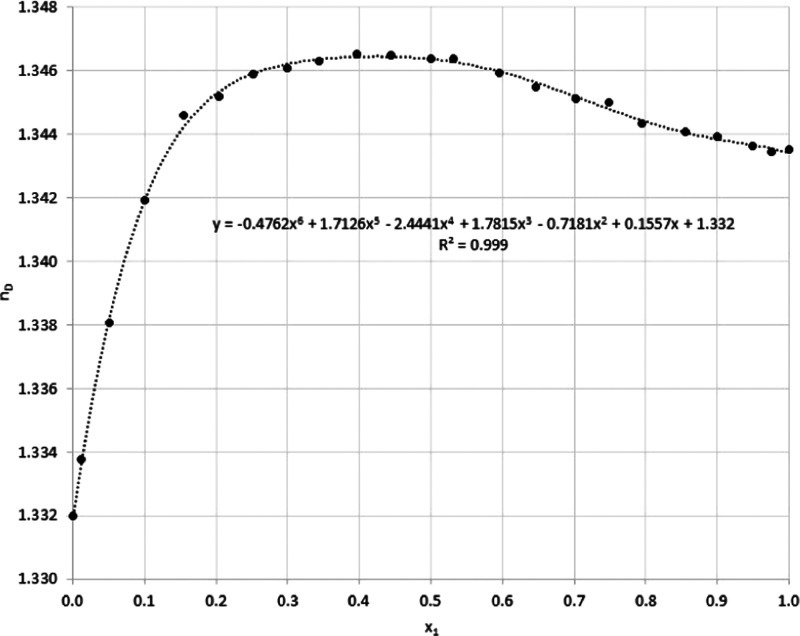
Experimental refractive indexes of the acetonitrile (1)–water (2) system at *T* = 293.2 K (●). *x*_1_: mole fraction of acetonitrile.

First, the modified equipment and VLE measurement procedure were validated with the acetonitrile–water binary mixture as a generally known and studied mixture. The refractive indexes were experimentally determined in the whole concentration range for acetonitrile (1)–water (2) mixture at *T* = 293.2 K. The data set are shown in Table 3. Figure 3 shows the concentration versus refractive index plots.

**Table 3:** Experimental Refractive Indexes (*n*_D_) of Acetonitrile (1)–Water (2) Mixture at 293.2 K, *P* = 101 kPa^a^

acetonitrile content		*n*_D_	acetonitrile content		*n*_D_
[mol/mol]	[g/g]	(293.15 K)	[mol/mol]	[g/g]	(293.15 K)
1.0000	1.0000	1.3434	0.4433	0.6447	1.3465
0.9753	0.9890	1.3436	0.3967	0.5998	1.3463
0.9496	0.9772	1.3439	0.3429	0.5432	1.3461
0.8996	0.9533	1.3441	0.3000	0.4941	1.3459
0.8545	0.9305	1.3443	0.2519	0.4341	1.3452
0.7942	0.8979	1.3450	0.2037	0.3682	1.3446
0.7492	0.8719	1.3451	0.1537	0.2927	1.3419
0.7020	0.8430	1.3455	0.1009	0.2036	1.3381
0.6466	0.8065	1.3459	0.0506	0.1083	1.3338
0.5961	0.7708	1.3464	0.0113	0.0254	1.3320
0.5317	0.7212	1.3464	0.0000	0.0000	1.3435
0.4994	0.6945	1.3465			

a Standard uncertainty *u* is *u*(*n*_D_) = 0.0001, *u*(*P*) = 2 kPa, and *u*(*T*) = 0.2 K.

Reis et al.^30^ demonstrated that the refractive index of thermodynamically ideal liquid mixtures can be expressed by the volume-fraction mixing rule of the pure-component squared refractive indices (Newton formula). This theoretical formulation entailed a positive change in refractive index upon ideal mixing, which was interpreted in terms of dissimilar London dispersion forces centered in the dissimilar molecules making up the mixture. For real liquid mixtures, the refractive index of mixing and the excess refractive index were introduced in a thermodynamic manner. Examples of mixtures were also cited for which excess refractive indices and excess molar volumes showed all of the four possible sign combinations, a fact that jeopardized the finding of a general equation linking these two excess properties. So far, there is no straightforward general explanation for this phenomenon for real liquid mixtures; therefore, we have collected numerical values for the refractive index at different decompositions of the acetonitrile–water binary system and fitted the sixth-order polynomial to the experimental points.

**Figure 4:**
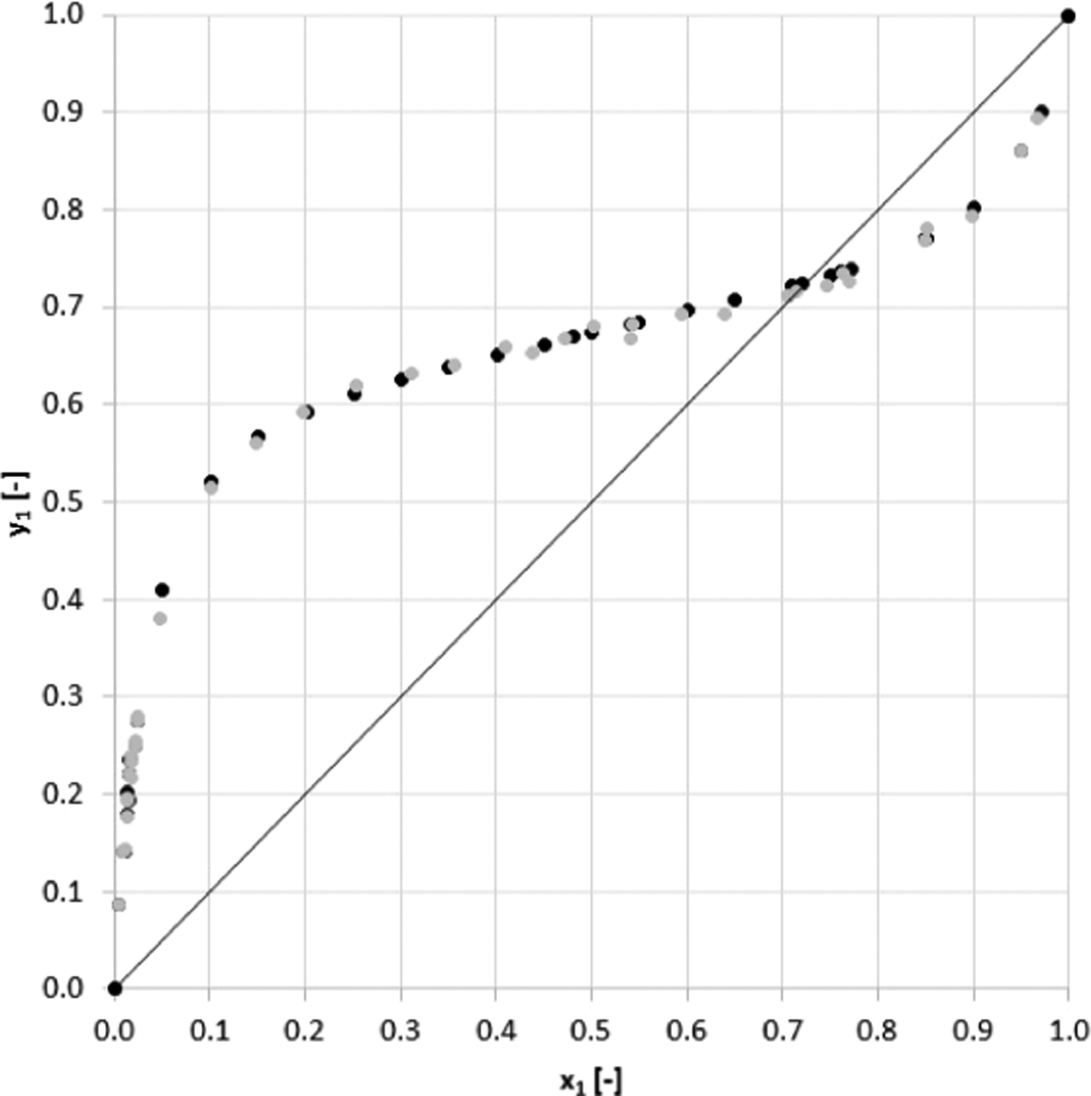
*y–x* Diagram for acetonitrile (1)–water (2) system at 101 kPa: (gray circle solid) experimental and (●) literature.

**Figure 5:**
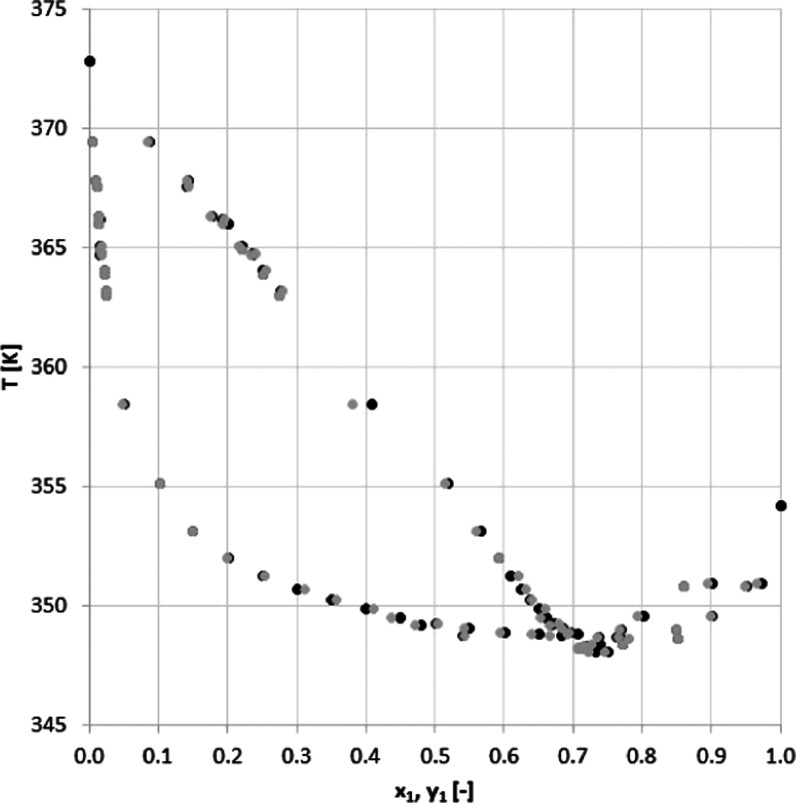
*T–y–x* diagram for acetonitrile (1)–water (2) system at 101 kPa: (gray circle solid) experimental and (●) literature.

As seen in Table 4 and Figures 4 and 5, the measured data reproduce all of the published VLE results excellently at 101 kPa.^31^ These measurements were carried out three times to provide information about the statistics of the measurement.

**Table 4:** Experimental Equilibrium Data for Acetonitrile (1)–Water (2) System at *P* = 101 kPa^a^

*T* [K]	*x*_1_	*y*_1_	*T* [K]	*x*_1_	*y*_1_
348.05	0.7460	0.7219	350.95	0.9670	0.8950
348.20	0.7069	0.7111	351.25	0.2536	0.6204
348.25	0.7070	0.7110	352.00	0.1985	0.5923
348.30	0.7150	0.7160	353.10	0.1498	0.5598
348.35	0.7710	0.7270	355.15	0.1013	0.5146
348.65	0.8510	0.7802	358.45	0.0483	0.3799
348.70	0.7645	0.7345	363.00	0.0248	0.2761
348.75	0.5420	0.6670	363.15	0.0252	0.2801
348.80	0.6403	0.6921	363.85	0.0218	0.2512
348.85	0.5945	0.6932	364.05	0.0220	0.2550
349.00	0.8487	0.7683	364.65	0.0180	0.2345
349.05	0.5432	0.6831	364.75	0.0182	0.2400
349.20	0.4720	0.6670	364.90	0.0167	0.2201
349.35	0.5034	0.6798	365.05	0.0174	0.2170
349.50	0.4380	0.6540	366.00	0.0148	0.1933
349.55	0.8987	0.7934	366.15	0.0146	0.1960
349.85	0.4103	0.6602	366.30	0.0130	0.1761
350.25	0.3567	0.6396	367.55	0.0122	0.1440
350.70	0.3127	0.6315	367.80	0.0100	0.1417
350.80	0.9498	0.8603	369.45	0.0057	0.0860

a In the case of experiments, the standard uncertainties *u* are *u*(*T*) = 0.1 K and *u*(*P*) = 2 kPa.

The relative average absolute deviation between experimental and extended literature values^31^ is 1.52% (*x*_1_) and 1.28% (*y*_1_).

**Figure 6:**
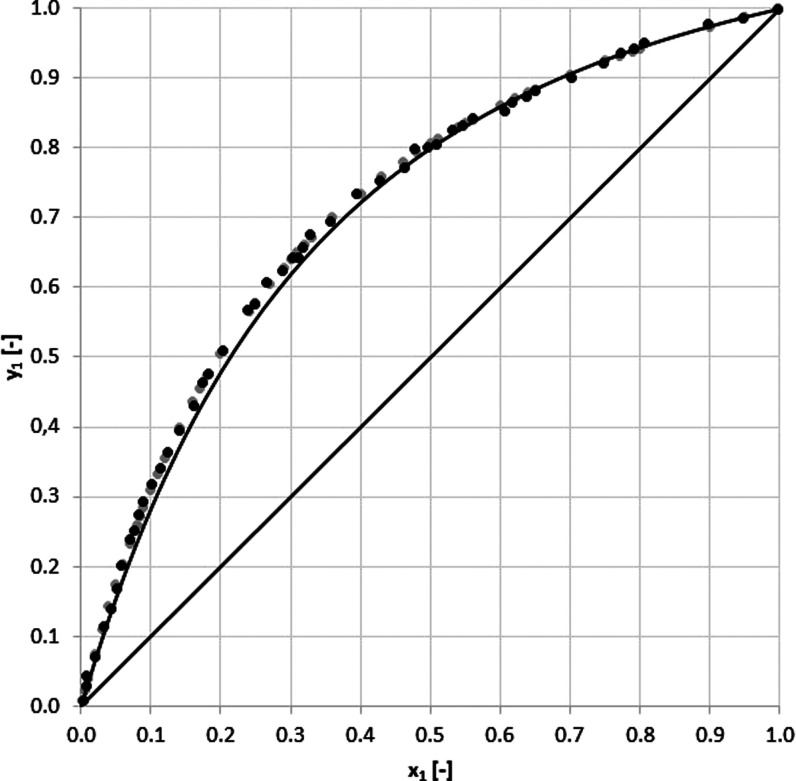
*y–x* Diagram for the monochlorobenzene (1)–4,6-dichloropyrimidine (2) system at *P* = 101 kPa with the experimental data (●), COSMO-SAC data (gray circle solid), and UNIFAC model (−).

**Figure 7:**
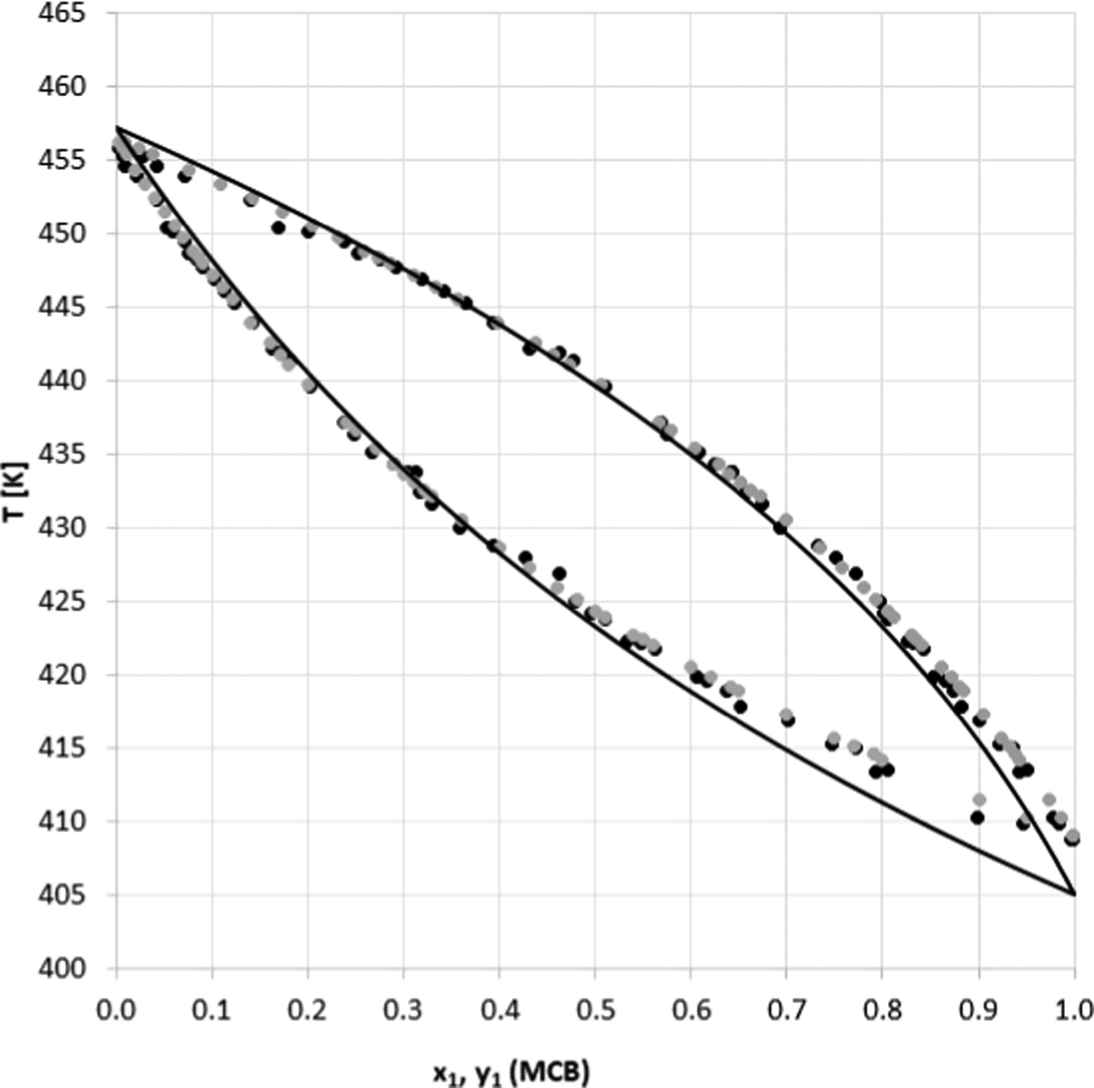
*T–y–x* diagram for the monochlorobenzene (1)–4,6-dichloropyrimidine (2) system at *P* = 101 kPa with the experimental data (●), COSMO-SAC data (gray circle solid), and UNIFAC model (−).

Since experimental information on the variation of the heat of mixing with temperature and composition is rarely available. The thermodynamic consistency test for the acetonitrile–water data was performed according to Herrington’s area test for isobaric data.^32^*D*_H_ and *J*_H_ values are calculated according to the following equations.^17^
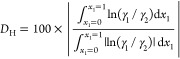
1
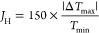
2
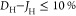
3The *D*_H_ value was 0.37% and the *J*_H_ value was found to be 7.60%; therefore, *D*_H_–*J*_H_ is 7.2%. It can be stated that the measured values are consistent and match with the literature data; therefore, our experimental setup can also provide accurate and reproducible data describing the isobaric vapor–liquid equilibrium of monochlorobenzene (1)–4,6-dichloropyrimidine (2) at *P* = 101 kPa. The MCB-DCP experimental and calculated data are presented in Tables 5–7 and Figures 6 and 7. The experiment verification can be taken with the objective function (OF), which showed a minimal deviation in the COSMO-SAC and the experiment values.
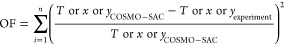
4Correlation between the OF values and the *x*_1_ values tabulated in Tables 6 and 7 has been observed. The OF values are a power function of the mole fraction in such a way that the OF value increases as *x*_1_ approaches 0. The average OFs are 9.9 × 10^–4^ (*T*), 6.3 × 10^–3^ (*x*_1_), and 1.7 × 10^–3^ (*y*_1_), and the corresponding standard deviations are 7.0 × 10^–3^ (*T*), 3.5 × 10^–2^ (*x*_1_), and 7.1 × 10^–3^ (*y*_1_). Standard deviation was calculated using the formula
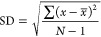
5where *x* takes each value in the set. *x̅* is the average of the set of values. *N* is the number of values.

**Table 5:** Comparison of Experimental VLE Data (*T*, Temperature) of Monochlorobenzene (1)–4,6-Dichloropyrimidine (2) at *P* = 101 kPa^a^ with COSMO-SAC VLE Data (*T*, Temperature)

experiment	COSMO-SAC	OF	experiment	COSMO-SAC	OF
*T* [K]	*T* [K]	*T* [K]	*T* [K]	*T* [K]	*T* [K]
408.85	409.06	2.7 × 10^–7^	433.81	433.74	2.4 × 10^–8^
409.89	410.26	8.1 × 10^–7^	434.38	434.30	3.6 × 10^–8^
410.35	411.54	8.4 × 10^–6^	435.15	435.44	4.4 × 10^–7^
413.56	414.27	3.0 × 10^–6^	436.45	436.62	1.6 × 10^–7^
413.34	414.56	8.6 × 10^–6^	437.18	437.23	1.3 × 10^–8^
414.98	415.14	1.4 × 10^–7^	439.65	439.79	9.5 × 10^–8^
415.34	415.73	8.8 × 10^–7^	441.34	441.14	2.0 × 10^–7^
416.87	417.26	8.8 × 10^–7^	441.89	441.84	1.3 × 10^–8^
417.89	418.88	5.6 × 10^–6^	442.23	442.55	5.4 × 10^–7^
418.89	419.21	5.9 × 10^–7^	443.98	444.03	1.2 × 10^–8^
419.67	419.89	2.8 × 10^–7^	445.34	445.57	2.6 × 10^–7^
419.87	420.59	2.9 × 10^–6^	446.12	446.36	3.0 × 10^–7^
421.80	422.04	3.2 × 10^–7^	446.98	447.18	1.9 × 10^–7^
422.12	422.41	4.7 × 10^–7^	447.76	448.01	3.1 × 10^–7^
422.35	422.79	1.1 × 10^–6^	448.32	448.44	6.7 × 10^–8^
423.87	423.96	4.1 × 10^–8^	448.65	448.86	2.2 × 10^–7^
424.23	424.35	8.6 × 10^–8^	449.56	449.74	1.5 × 10^–7^
425.09	425.17	3.6 × 10^–8^	450.23	450.63	7.9 × 10^–7^
426.87	426.01	4.1 × 10^–6^	450.45	451.55	5.9 × 10^–6^
427.98	427.32	2.4 × 10^–6^	452.31	452.48	1.5 × 10^–7^
428.75	428.68	2.6 × 10^–8^	352.53	453.45	5.0 × 10^–2^
430.03	430.61	1.8 × 10^–6^	453.98	454.44	1.0 × 10^–6^
431.65	432.13	1.3 × 10^–6^	454.63	455.45	3.2 × 10^–6^
432.42	432.66	3.1 × 10^–7^	455.32	455.86	1.4 × 10^–6^
433.76	433.20	1.7 × 10^–6^	455.87	456.28	8.0 × 10^–7^

a In the case of experiments, the standard uncertainties *u* are *u*(T) = 0.1 K and *u*(*P*) = 2 kPa.

**Table 6:** Comparison of Experimental VLE Data (*x*, Liquid Mole Fraction) of Monochlorobenzene (1)–4,6-Dichloropyrimidine (2) at *P* = 101 kPa^a^ with COSMO-SAC VLE Data (*x*, Liquid Mole Fraction)

experiment	COSMO-SAC	OF	experiment	COSMO-SAC	OF
*x*_1_	*x*_1_	*x*_1_	*x*_1_	*x*_1_	*x*_1_
0.997	0.998	1.0 × 10^–6^	0.303	0.300	1.0 × 10^–4^
0.947	0.950	1.0 × 10^–5^	0.289	0.290	1.2 × 10^–5^
0.898	0.900	4.9 × 10^–6^	0.266	0.270	2.2 × 10^–4^
0.806	0.800	5.6 × 10^–5^	0.248	0.250	6.4 × 10^–5^
0.792	0.790	6.4 × 10^–6^	0.238	0.240	6.9 × 10^–5^
0.773	0.770	1.5 × 10^–5^	0.203	0.200	2.3 × 10^–4^
0.747	0.750	1.6 × 10^–5^	0.182	0.180	1.2 × 10^–4^
0.702	0.700	8.2 × 10^–6^	0.174	0.170	5.5 × 10^–4^
0.651	0.650	2.4 × 10^–6^	0.162	0.160	1.6 × 10^–4^
0.637	0.640	2.2 × 10^–5^	0.141	0.140	5.1 × 10^–5^
0.617	0.620	2.3 × 10^–5^	0.124	0.120	1.1 × 10^–3^
0.606	0.600	1.0 × 10^–4^	0.113	0.110	7.4 × 10^–4^
0.561	0.560	3.2 × 10^–6^	0.102	0.100	4.0 × 10^–4^
0.547	0.550	3.0 × 10^–5^	0.089	0.090	1.2 × 10^–4^
0.532	0.540	2.2 × 10^–4^	0.083	0.085	5.5 × 10^–4^
0.509	0.510	3.8 × 10^–6^	0.076	0.080	2.5 × 10^–3^
0.496	0.500	6.4 × 10^–5^	0.071	0.070	2.0 × 10^–4^
0.478	0.480	1.7 × 10^–5^	0.058	0.060	1.1 × 10^–3^
0.463	0.460	4.3 × 10^–5^	0.052	0.050	1.6 × 10^–3^
0.427	0.430	4.9 × 10^–5^	0.043	0.040	5.6 × 10^–3^
0.394	0.400	2.3 × 10^–4^	0.033	0.030	1.0 × 10^–2^
0.358	0.360	3.1 × 10^–5^	0.021	0.020	2.5 × 10^–3^
0.328	0.330	3.7 × 10^–5^	0.009	0.010	1.0 × 10^–2^
0.317	0.320	8.8 × 10^–5^	0.007	0.006	2.8 × 10^–2^
0.312	0.310	4.2 × 10^–5^	0.003	0.002	2.5 × 10^–1^

a In the case of experiments, the standard uncertainties *u* are *u*(*T*) = 0.1 K and *u*(*P*) = 2 kPa.

**Table 7:** Comparison of Experimental VLE Data (*y*, Vapor Mole Fraction) of Monochlorobenzene (1)–4,6-Dichloropyrimidine (2) at *P* = 101 kPa^a^ with COSMO-SAC VLE Data (*y*, Vapor Mole Fraction)

experiment	COSMO-SAC	OF	experiment	COSMO-SAC	OF
*y*_1_	*y*_1_	*y*_1_	*y*_1_	*y*_1_	*y*_1_
0.998	0.999	2.2 × 10^–6^	0.642	0.639	1.5 × 10^–5^
0.985	0.987	3.5 × 10^–6^	0.624	0.628	4.4 × 10^–5^
0.978	0.973	2.7 × 10^–5^	0.608	0.604	3.7 × 10^–5^
0.951	0.942	9.3 × 10^–5^	0.575	0.579	4.3 × 10^–5^
0.942	0.939	1.3 × 10^–5^	0.568	0.565	2.3 × 10^–5^
0.936	0.932	2.2 × 10^–5^	0.509	0.506	3.1 × 10^–5^
0.921	0.925	1.4 × 10^–5^	0.476	0.473	3.9 × 10^–5^
0.901	0.905	2.5 × 10^–5^	0.463	0.455	2.7 × 10^–4^
0.882	0.885	8.2 × 10^–6^	0.431	0.437	2.0 × 10^–4^
0.873	0.880	6.5 × 10^–5^	0.394	0.398	1.1 × 10^–4^
0.865	0.871	4.6 × 10^–5^	0.364	0.356	5.2 × 10^–4^
0.853	0.861	9.3 × 10^–5^	0.341	0.333	5.3 × 10^–4^
0.843	0.841	7.0 × 10^–6^	0.318	0.310	7.2 × 10^–4^
0.832	0.835	1.6 × 10^–5^	0.292	0.285	6.1 × 10^–4^
0.826	0.830	2.1 × 10^–5^	0.275	0.272	1.2 × 10^–4^
0.805	0.812	7.9 × 10^–5^	0.251	0.259	9.8 × 10^–4^
0.801	0.806	4.0 × 10^–5^	0.238	0.232	6.7 × 10^–4^
0.798	0.793	3.5 × 10^–5^	0.201	0.204	1.6 × 10^–4^
0.772	0.780	1.0 × 10^–4^	0.168	0.174	1.1 × 10^–3^
0.752	0.758	6.9 × 10^–5^	0.139	0.142	5.9 × 10^–4^
0.733	0.735	6.4 × 10^–6^	0.114	0.110	1.7 × 10^–3^
0.694	0.700	7.9 × 10^–5^	0.071	0.075	2.7 × 10^–3^
0.675	0.671	3.0 × 10^–5^	0.043	0.038	1.4 × 10^–2^
0.658	0.661	2.1 × 10^–5^	0.028	0.023	4.7 × 10^–2^
0.643	0.650	1.3 × 10^–4^	0.007	0.008	1.2 × 10^–2^

a In the case of experiments, the standard uncertainties *u* are *u*(*T*) = 0.1 K and *u*(*P*) = 2 kPa.

It can be stated that no azeotropic mixture was formed from monochlorobenzene (1)–4,6-dichloropyrimidine (2) in the investigated concentration range, and the COSMO-SAC program is capable of the VLE data set description. According to the *y*_1_(*x*_1_) diagram shown in Figure 6, the experiment and the two models seem to be consistent, and the increased discrepancy can be only observed in the range of 0.05 < *x*_1_ < 0.4 for UNIFAC-based estimates. On the other hand, a larger deviation in the UNIFAC-based estimates for the *T*(*x*_1_, *y*_1_) curve compared to the COSMO-SAC results can be seen in Figure 7. To conclude, the COSMO-SAC data has more accurate temperature tracking, confirming that models always need to be refined.

The measured data of the monochlorobenzene–4,6-dichloropyrimidine binary mixture are considered consistent according to Herington’s consistency test. The *D*_H_ value is 10.96% and the *J*_H_ value is found to be 16.29%; therefore, *D*_H_–*J*_H_ is 5.3%. Furthermore, two other mixtures are also investigated with the experimental VLE apparatus. The measured VLE data of the acetal–ethanol and acetaldehyde–ethanol binary mixtures are consistent too.^17^

## 4 Conclusions

To satisfy the urgent need for reliable physicochemical data of rare and/or new chemical vapor–liquid equilibrium (VLE) data for the monochlorobenzene (1)–4,6-dichloropyrimidine (2) binary system were measured at atmospheric (101 kPa) pressure using a modified Gillespie still. It was demonstrated that 4,6-dichloropyrimidine could be completely separated from monochlorobenzene without the formation of an azeotropic mixture. The VLE data were also calculated with the COSMO-SAC using the ADF software. The calculation and measurement of the data showed reliable physicochemical data. It must be mentioned that there was consistency between the results of COSMO-SAC calculations and VLE experiments. The results can encourage chemical engineers to apply calculation methods to make up missing physicochemical data urgently needed by the process design.

## Supporting Information Available

The conditions of GC analytics: chromatogram of the MCB-DCP sample (taken by Andras Jozsef Toth. Copyright 2022) (Figure S1); chromatogram of the MCB-DCP sample (taken by Andras Jozsef Toth. Copyright 2022) (Figure S2); chromatogram of the MCB-DCP sample (taken by Andras Jozsef Toth. Copyright 2022) (Figure S3); chromatogram of the MCB-DCP sample (taken by Andras Jozsef Toth. Copyright 2022) (Figure S4); and chromatogram of the MCB-DCP sample (taken by Andras Jozsef Toth. Copyright 2022) (Figure S5) (PDF)
