# Supplementary material for: Vapor–Liquid Equilibrium Study of the Monochlorobenzene–4,6-Dichloropyrimidine Binary System
Source: ACS Omega. 2022 May 19;7(21):17670–8. doi: 10.1021/acsomega.2c00525 (PMC9161255; doi:10.1021/acsomega.2c00525)
Supplement: Supplementary file 1 — ao2c00525_si_001.pdf [file ao2c00525_si_001.pdf]

Supporting Information

Vapor−Liquid Equilibrium Study of the Monochlorobenzene–4,6-Dichloropyrimidine Binary System

Eniko Haaz[1], Daniel Fozer[2], Ravikumar Thangaraj[3,4], Milán Szőri[3], Peter Mizsey[3], Andras Jozsef Toth*[,1]

[1]Environmental and Process Engineering Research Group, Department of Chemical and Environmental Process Engineering, Budapest University of Technology and Economics, H-1111, Hungary, Budapest, Műegyetem rkp. 3.

[2]Division for Sustainability, Department of Environmental and Resource Engineering, Technical University of Denmark, Produktionstorvet, Building, 424, DK-2800 Kgs. Lyngby, Denmark

[3]Institute of Chemistry, Faculty of Material Science and Engineering, University of Miskolc, H-3515, Hungary, Miskolc, Egyetemváros A/2.

[4]Higher Education and Industry Cooperation Center of Advanced Materials and Intelligent Technologies, University of Miskolc, H-3515, Hungary, Miskolc, Egyetemváros A/2.

* Corresponding author. E-mail address: andrasjozseftoth@edu.bme.hu, Tel: +36 1 463 1490; Fax: +36 1 463 3197

Chromatographic conditions

Instrument:	GC2010Plus+AOC-20 autosampler gas chromatograph
Column:	HP-5 (30 m x 0.32 mm, 0.25 µm)
Detector (FID):	H_2 flow: 30 ml/min
	Air flow: 350 ml/min
	N_2 flow: 25 ml/min
Oven Program:	40 °C for 1 min then 15 °C/min to 220 °C for 2 min.
Heater:	Detector: 280 °C
	Injector: 250 °C
Carrier Gas:	H_2
Make up Gas:	N_2
Injection volume:	1.2 µl
Split:	30:1
Run time:	15 min
Diluent:	Dichloromethane

Standard solution:

The concentration of 4,6-Dichloropyrimidine (DCP) and Monochlorobenzene (MCB) should be chosen according to the expected concentration in the samples. The solution can be used at least for 1 year if stored at 2-8 °C.

Suggestion:

Weigh accurately 200 mg of DCP reference material and 800 mg of MCB reference material into a 10 ml volumetric flask, then fill up to volume with diluent, homogenize it in ultrasonic bath for 5 minutes, then shake well. Prepare two standard solutions, and inject 3 times from each for the calibration.

Sample solution:

Weigh accurately 100 mg of DCP solid sample into a 1.0 ml volumetric flask, fill up to volume with diluent, homogenize it in ultrasonic bath for 5 minutes, then shake well (c ~ 100000 µg/ml DCP).

In case of liquid samples pipette 100 µl of DCP sample into a 1.0 ml volumetric flask, fill up to volume with diluent, then shake well.

Blank solution: Diluent.

Procedure:

Inject the blank solution, the standard solutions and the sample solutions.

Informative retention time

	**RT**
DCM	1.1 min
MCB	3.1 min
**DCP**	**4.8 min**

Evaluation

Concentration of the components is determined via external standard method and is given by the following formula:

$$\text{Concentration (\%)} = \frac{A_{sample} \cdot C_{std} \cdot P_{std}}{A_{std} \cdot C_{sample}}$$, where:

A_{sample}	Area of analyte peak in the chromatogram of the sample solution
A_{std}	Area of analyte peak in the chromatogram of the standard solution
C_{sample}	Concentration of analyte in the sample solution (µg/ml)
C_{std}	Concentration of analyte in the standard solution (µg/ml)
P_{std}	Potency of standard material (%)

Typical chromatograms of sample solution can be seen in Figure S1-S5.

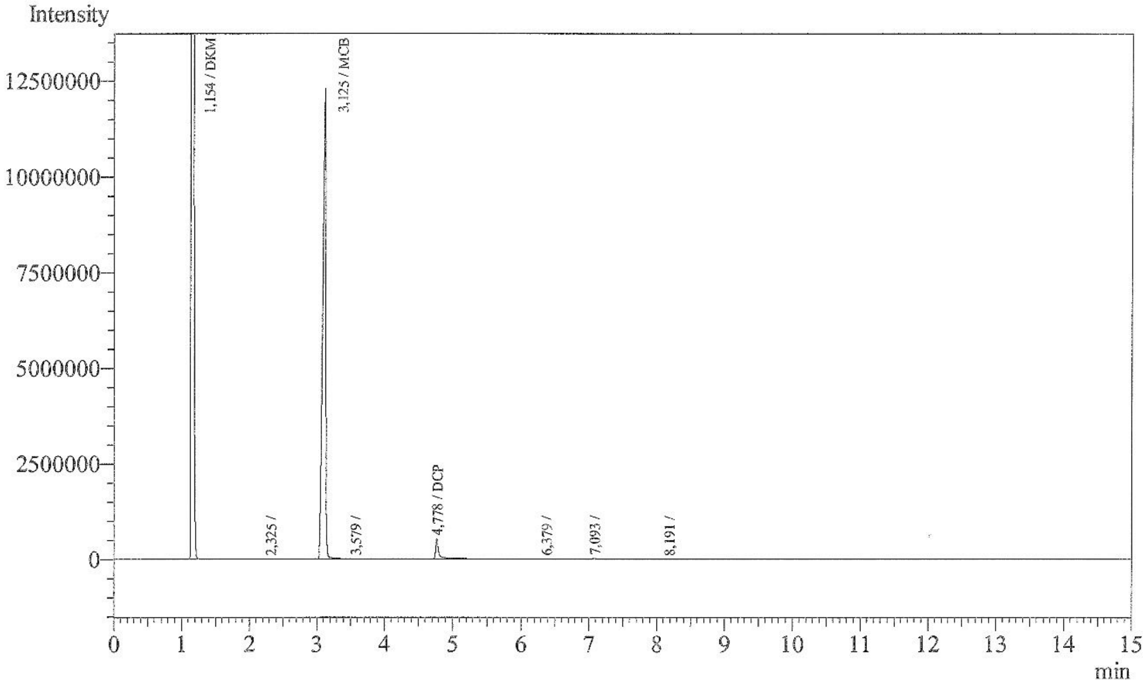

Peak#	Ret.Time	Area	Height	Conc.	Unit	Mark	ID#	Cmpd Name
1	1,154	92556172	40612317	0,000	ppm	SV	1	DKM
2	2,325	1234	481	0,000		T		
3	3,125	38492364	12198627	0,000	ppm	S	2	MCB
4	3,579	2853	1518	0,000		T		
5	4,778	2009411	532777	0,000	ppm	S	3	DCP
6	6,379	1122	408	0,000		T		
7	7,093	74634	22057	0,000		T		
8	8,191	1144	42	0,000		TV		
Total		133138934	53368227					

Figure S1. Chromatogram of MCB-DCP sample (Taken by Andras Jozsef Toth. Copyright 2022).

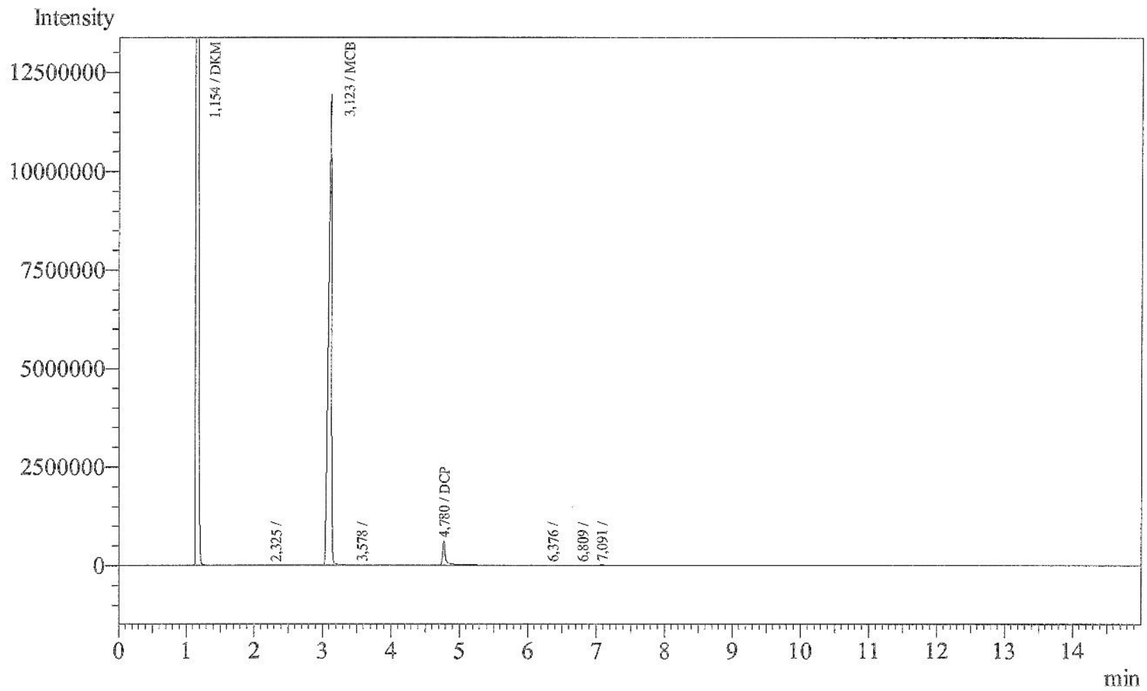

Peak#	Ret.Time	Area	Height	Conc.	Unit	Mark	ID#	Cmpd Name
1	1,154	91234627	40292434	0,000	ppm	SV	1	DKM
2	2,325	1006	392	0,000		T		
3	3,123	37135703	11857853	0,000	ppm	S	2	MCB
4	3,578	2799	1508	0,000		T		
5	4,780	2181907	605000	0,000	ppm	T	3	DCP
6	6,376	41755	2144	0,000		TV		
7	6,809	13343	1056	0,000		TV		
8	7,091	101613	22856	0,000		TV		
Total		130712753	52783243					

Figure S2. Chromatogram of MCB-DCP sample (Taken by Andras Jozsef Toth. Copyright 2022).

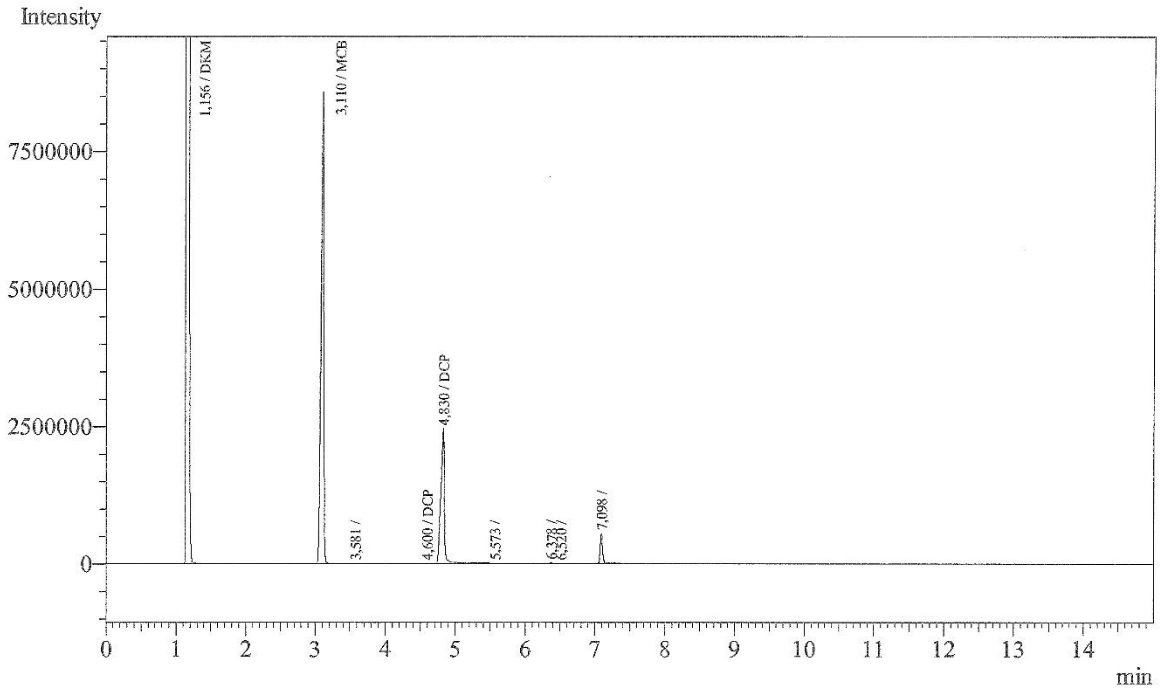

Peak#	Ret.Time	Area	Height	Conc.	Unit	Mark	ID#	Cmpd Name
1	1,156	90998911	40768298	0,000	ppm	S	1	DKM
2	3,110	22805499	8501181	0,000	ppm	S	2	MCB
3	3,581	3773	1638	0,000		T		
4	4,600	1455	555	0,000	ppm		3	DCP
5	4,830	8656557	2410367	0,000	ppm	S	3	DCP
6	5,573	1015	523	0,000		T		
7	6,378	18645	7588	0,000		T		
8	6,520	1185	294	0,000		TV		
9	7,098	1411525	536410	0,000		SV		
Total		123898565	52226854					

Figure S3. Chromatogram of MCB-DCP sample (Taken by Andras Jozsef Toth. Copyright 2022).

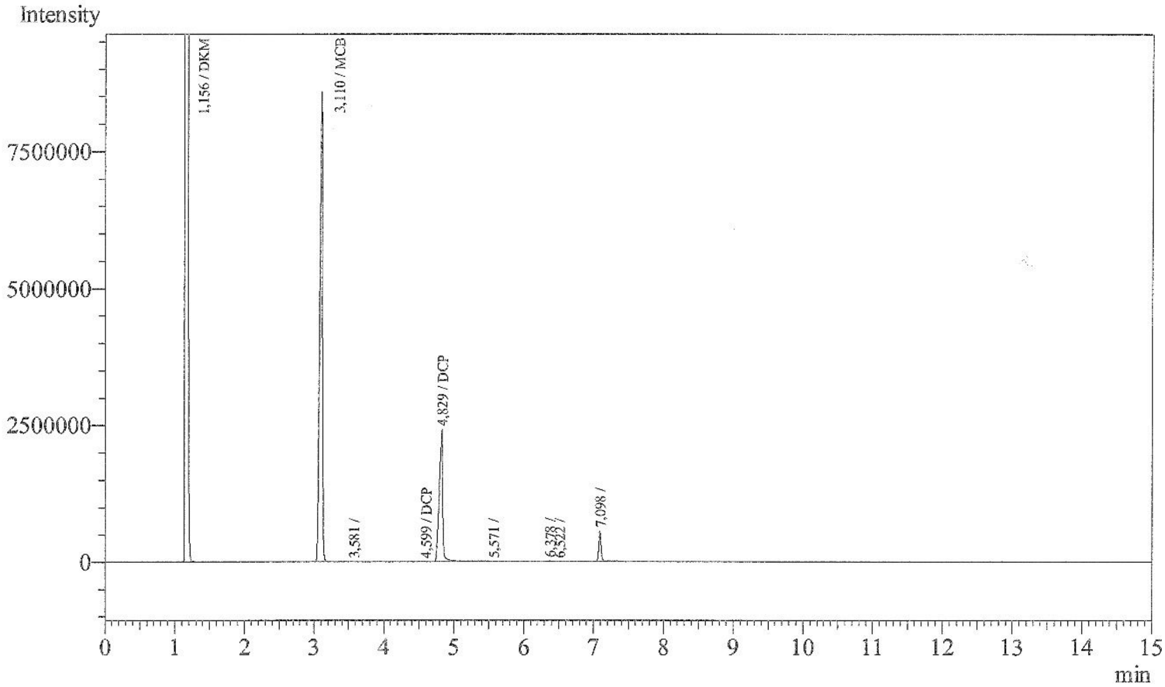

Peak#	Ret.Time	Area	Height	Conc.	Unit	Mark	ID#	Cmpd Name
1	1,156	92172270	41138296	0,000	ppm	S	1	DKM
2	3,110	22516899	8523140	0,000	ppm	S	2	MCB
3	3,581	3734	1647	0,000		T		
4	4,599	1544	592	0,000	ppm		3	DCP
5	4,829	8660169	2397561	0,000	ppm	SV	3	DCP
6	5,571	1081	480	0,000		T		
7	6,378	19040	7761	0,000		T		
8	6,522	1054	297	0,000		TV		
9	7,098	1460777	556130	0,000		SV		
Total		124836568	52625904					

Figure S4. Chromatogram of MCB-DCP sample (Taken by Andras Jozsef Toth. Copyright 2022).

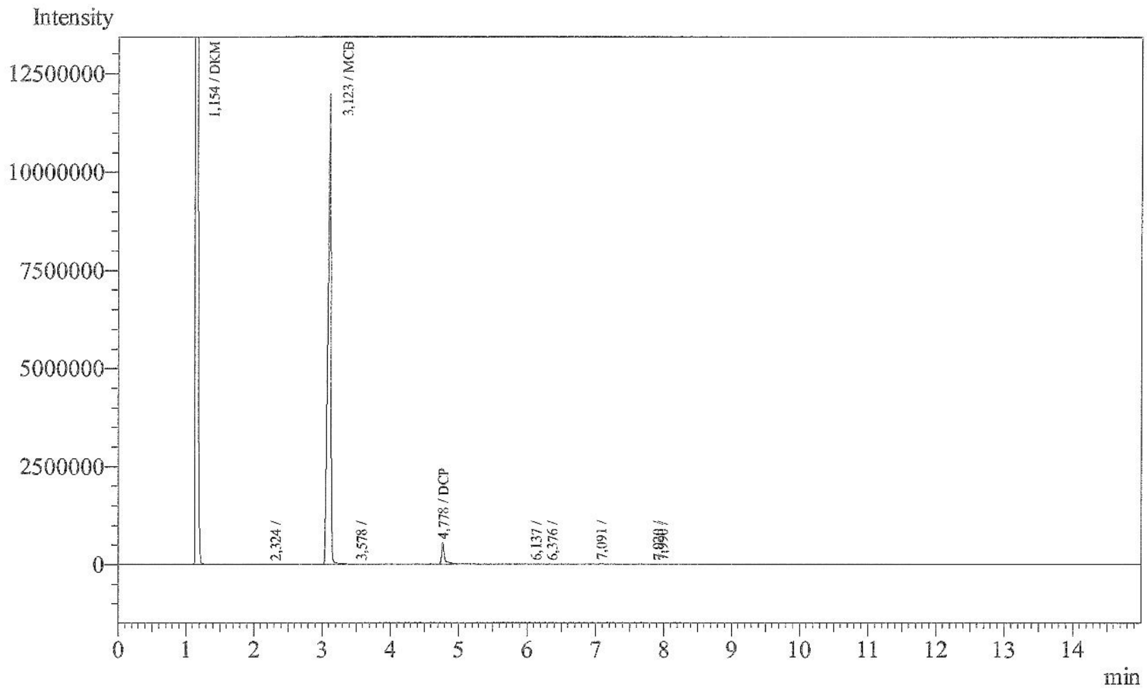

Peak#	Ret.Time	Area	Height	Conc.	Unit	Mark	ID#	Cmpd Name
1	1,154	91150437	40433076	0,000	ppm	SV	1	DKM
2	2,324	1194	437	0,000		T		
3	3,123	36464290	11682914	0,000	ppm	S	2	MCB
4	3,578	2683	1466	0,000		T		
5	4,778	1959230	547877	0,000	ppm	T	3	DCP
6	6,137	23483	2126	0,000		TV		
7	6,376	49424	1998	0,000		TV		
8	7,091	89325	20554	0,000		TV		
9	7,920	1249	252	0,000		TV		
10	7,990	1745	199	0,000		TV		
Total		129743060	52690899					

Figure S5. Chromatogram of MCB-DCP sample (Taken by Andras Jozsef Toth. Copyright 2022).
